# Artificial Intelligence in Esophagectomy: A Systematic Review

**DOI:** 10.3390/jcm15062169

**Published:** 2026-03-12

**Authors:** Vladimir Aleksiev, Daniel Markov, Kristian Bechev, Desislav Stanchev, Filip Shterev, Galabin Markov

**Affiliations:** 1 Department of Cardiovascular Surgery, Medical University of Plovdiv, 4002 Plovdiv, Bulgaria; 2 Department of Thoracic Surgery, University Hospital “Kaspela”, 4002 Plovdiv, Bulgaria; filip.shterev@mu-plovdiv.bg; 3 Department of General and Clinical Pathology, Medical University of Plovdiv, 4002 Plovdiv, Bulgaria; daniel.markov@mu-plovdiv.bg; 4 Department of Clinical Pathology, University Multidisciplinary Hospital for Active Treatment “Pulmed”, 4002 Plovdiv, Bulgaria; 5 Department of Neurological Surgery, Pulmed University Hospital, 4000 Plovdiv, Bulgaria; kristian.bechev@mu-plovdiv.bg; 6 II-nd Department of Internal Diseases, Section of Gastroenterology, Medical University of Plovdiv, 4002 Plovdiv, Bulgaria; desislav.stanchev@mu-plovdiv.bg; 7 Department of Gastroenterology, University Hospital “Kaspela”, 4002 Plovdiv, Bulgaria; 8 I-st Department of Internal Diseases, Section of Pneumology and Phthysiatrics, Medical University of Plovdiv, 4002 Plovdiv, Bulgaria; 9 Faculty of Medicine, Medical University of Plovdiv, 4002 Plovdiv, Bulgaria; gabi_markov@abv.bg

**Keywords:** esophagectomy, artificial intelligence, intraoperative video analysis, minimally invasive surgery, robotic surgery

## Abstract

**Background**: Esophagectomy remains a technically demanding oncologic procedure with substantial morbidity, despite ongoing advances in minimally invasive and robotic techniques. Limitations in intraoperative visualization and anatomical recognition contribute to complications such as nerve injury and bleeding. Artificial intelligence (AI)-based intraoperative video analysis has emerged as a potential adjunct to enhance surgical perception and safety, but its application in esophagectomy has not been comprehensively reviewed. **Methods**: A systematic review was conducted in accordance with PRISMA guidelines. PubMed, Scopus, and Web of Science were searched without a lower date limit to identify eligible studies published up to January 2026, capturing early and contemporary applications of intraoperative AI in esophagectomy. Human studies involving any surgical approach were included. Data on the AI task, methodology, validation strategy, performance metrics, and reported clinical outcomes was extracted. Risk of bias was assessed using the ROBINS-I tool. **Results**: Six studies met the inclusion criteria, predominantly evaluating AI-driven analysis of intraoperative video during minimally invasive or robotic esophagectomy. Reported applications included real-time anatomical structure recognition, recurrent laryngeal nerve segmentation, detection of excessive nerve traction, instrument and event recognition, and surgical phase identification. Across studies, AI systems demonstrated performance comparable to expert surgeons for selected tasks and achieved real-time or near–real-time inference. One study reported earlier detection of excessive recurrent laryngeal nerve traction compared to conventional nerve integrity monitoring. However, most studies were retrospective, single-center, and feasibility-focused, with limited external validation and minimal assessment of patient-centered clinical outcomes. **Conclusions**: Artificial intelligence-based intraoperative analysis in esophagectomy is increasingly achievable and may enhance anatomical recognition, intraoperative risk detection, and procedural awareness. Nevertheless, current evidence remains preliminary, heterogeneous, and largely exploratory. Prospective, multicenter studies with standardized reporting and clinically meaningful outcome evaluation are required before routine implementation. Until such data is available, AI should be regarded as a complementary intraoperative tool rather than a standalone clinical decision-making system.

## 1. Introduction

Despite the emerging advances in surgical oncology, esophageal cancer remains the 7th leading cause of cancer death worldwide [1]. It ranks among the leading causes of malignancy-related mortality, accounting for nearly 500,000 deaths each year, with just as many people being diagnosed with the disease annually [2]. Its aggressive biological behavior, tendency for early lymphatic spread, and frequent late presentation contribute to a poor prognosis and a 5-year-survival rate of less than 25% [3]. For patients with potentially curable disease, multimodal treatment consisting of neoadjuvant chemoradiotherapy or chemotherapy followed by surgical resection has become the standard of care and offers the best chance for long-term survival [4].

Esophagectomy, however, is among the most complex procedures in surgery. The operation requires meticulous dissection within anatomically confined and highly vascularized regions, including the mediastinum and abdomen, in close proximity to vital structures such as the recurrent laryngeal nerve, thoracic duct, major vessels, and airway. Limited exposure, challenging visualization, and demanding ergonomics contribute to a steep learning curve and a substantial risk of perioperative morbidity, even in experienced centers [5]. As a result, complications such as anastomotic leakage, pulmonary morbidity, and nerve injury remain prevalent and significantly impact postoperative outcomes [6].

The introduction of minimally invasive esophagectomy (MIE), including video-assisted thoracoscopic and laparoscopic approaches, has aimed to mitigate surgical trauma while maintaining oncologic radicality. More recently, robotic-assisted minimally invasive esophagectomy (RAMIE) has further transformed the surgical landscape by offering high-definition three-dimensional visualization, instrument articulation, tremor filtration, and improved surgeon ergonomics [7]. These advantages enhance exposure and precision during complex mediastinal dissection. Despite these technological developments, esophagectomy continues to be associated with substantial complication rates ranging from 20 to 40% across different surgical approaches [8]. This suggests that limitations in intraoperative perception, anatomy identification, and spatial awareness remain key contributors to adverse outcomes.

**Figure 1:**
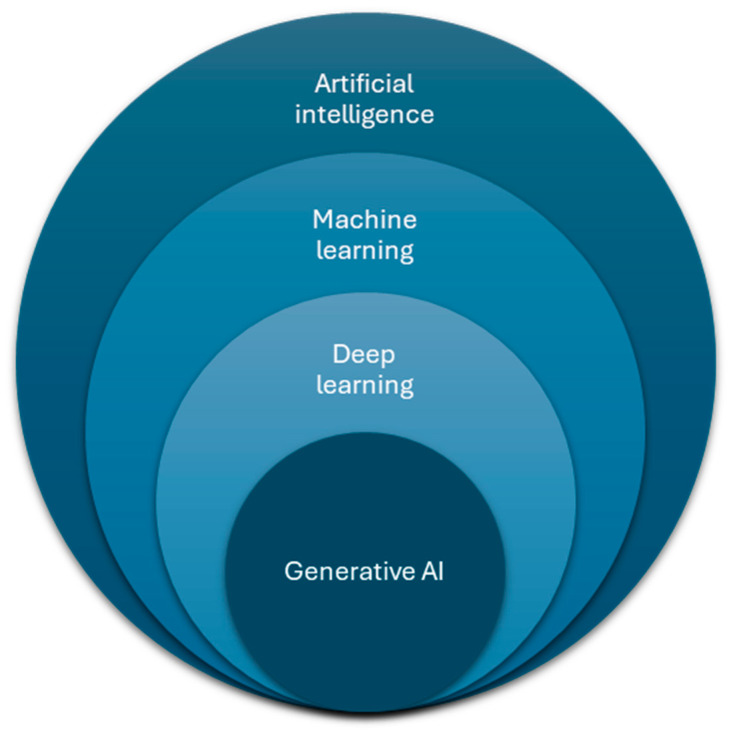
Conceptual hierarchy of artificial intelligence, illustrating the relationship between broad AI, machine learning, deep learning, and generative AI models.

Artificial intelligence (AI) has emerged as a promising tool to address such limitations. Broadly, AI encompasses computational techniques that enable machines to perform tasks traditionally requiring human intelligence. In healthcare and surgery, AI applications are typically categorized into rule-based systems, machine learning algorithms, and deep learning models. Deep learning approaches often rely on neural networks that extract complex patterns from large datasets [9] (Figure 1). The increasing availability of high-quality surgical video data and digital perioperative information has positioned surgery as a particularly suitable domain for AI-driven innovation.

**Figure 2:**
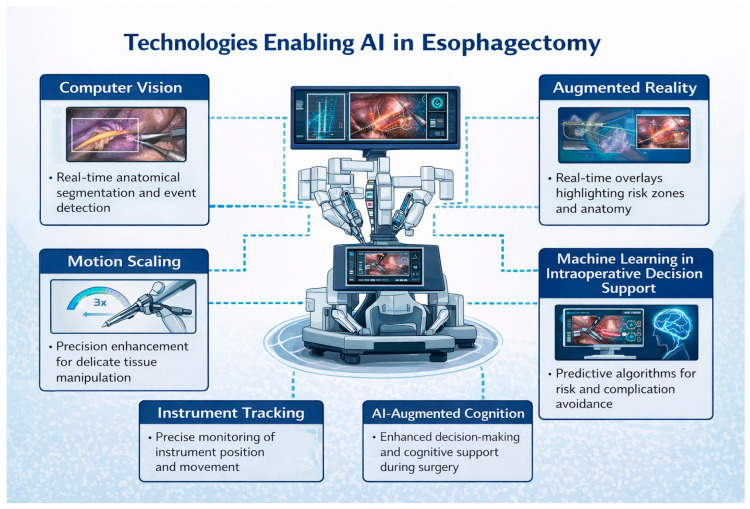
Conceptual framework of technologies enabling artificial intelligence integration during esophagectomy, including real-time segmentation, instrument tracking, and decision support.

In surgical practice, AI has been applied to a range of tasks, including intraoperative image and anatomy recognition, surgical phase and workflow analysis, performance assessment, and decision support. In visually intensive procedures such as minimally invasive and robotic esophagectomy, AI-based analysis of operative video holds particular promise for improving anatomical orientation, enhancing safety, and supporting surgeon decision-making [10] (Figure 2). Despite growing interest and an expanding body of literature, the role of AI in esophagectomy has not yet been comprehensively synthesized.

### Objective of the Study

The objective of this systematic review is to identify, summarize, and critically appraise the existing clinical and technical evidence on the intraoperative use of artificial intelligence during esophagectomy, with particular focus on the types of AI technologies employed, their intraoperative applications, validation methods, and reported clinical outcomes.

## 2. Materials and Methods

### 2.1. Study Design and Reporting Standards

**Figure 3:**
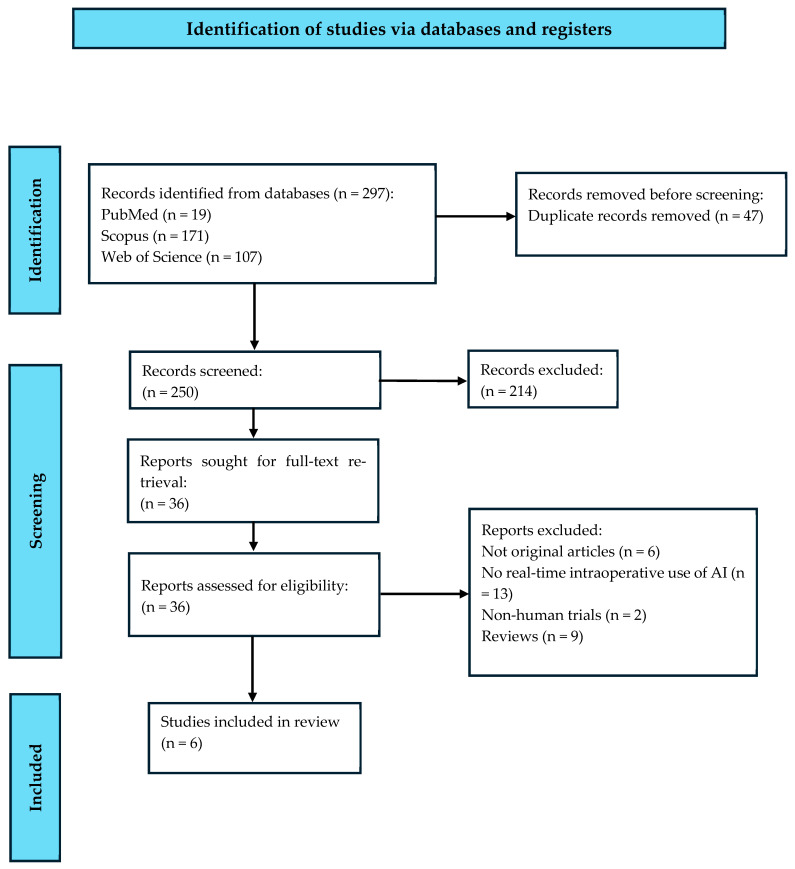
PRISMA flowchart outlining the systematic search process.

This systematic review was conducted and reported in accordance with the Preferred Reporting Items for Systematic Reviews and Meta-Analyses (PRISMA) 2020 statement [11]. The PRISMA checklist is provided in the Supplementary Materials (Figure 3). The review aimed to evaluate the current evidence on the intraoperative application of artificial intelligence in esophagectomy.

### 2.2. Review Question and PICO Framework

The review question was defined using the PICO framework. The population of interest consisted of adult patients undergoing esophagectomy using any surgical approach, including open, minimally invasive, hybrid, or robotic techniques. The intervention was the intraoperative use of artificial intelligence, including but not limited to machine learning, deep learning, computer vision, and decision-support systems capable of real-time or near–real-time computational processing. Studies with or without a comparator, including comparisons with conventional surgery without AI assistance, were eligible. Outcomes of interest included feasibility, technical accuracy, validation performance, intraoperative decision-making, safety, and reported clinical outcomes.

### 2.3. Literature Search Strategy

A systematic literature search was performed in PubMed, Scopus, and Web of Science to identify relevant studies reporting intraoperative applications of artificial intelligence in esophagectomy. The search was conducted without a predefined lower date limit in order to allow for a comprehensive capture of early intraoperative applications of artificial intelligence during esophagectomy. All eligible studies published up to January 2026 were included. The last search was conducted on 15 January 2026. The last search was conducted on 15 January 2026. The search strategy combined terms related to esophagectomy and artificial intelligence, with particular emphasis on intraoperative applications and AI-assisted surgical video analysis. The mentioned search strategies are reviewed in detail in the Supplementary Materials.

### 2.4. Eligibility Criteria

Studies were eligible for inclusion if they met the following criteria:

- Original studies reporting the intraoperative use of artificial intelligence;
- Procedures involving esophagectomy;
- Human studies;
- Any surgical approach, including open, minimally invasive, or robotic surgery;
- Reporting technical performance metrics and/or clinical outcomes.

Studies were excluded if they met any of the following criteria:

- Artificial intelligence used exclusively in the preoperative setting (e.g., imaging, staging, prediction of response to neoadjuvant therapy) or postoperative setting (e.g., prediction of complications);
- Simulation, phantom, cadaveric, or animal studies;
- Conference abstracts without available full text;
- Reviews, editorials, commentaries, or letters;
- Studies not involving esophageal surgery or focusing on other malignancies such as gastric cancer.

### 2.5. Study Selection

All records identified through the database searches were exported to a standardized spreadsheet, and duplicates were removed. Two reviewers independently screened titles and abstracts to exclude studies not involving artificial intelligence or not addressing its intraoperative use during esophagectomy. Full-text articles were then independently assessed for eligibility by the same reviewers based on the predefined inclusion and exclusion criteria. Any disagreements were resolved through discussion with a third reviewer. Studies focusing exclusively on preoperative or postoperative AI applications were excluded at this stage.

### 2.6. Data Extraction

Data was extracted independently by two reviewers into a standardized spreadsheet. Discrepancies were resolved by consensus or consultation with a third reviewer. Extracted variables included study characteristics, surgical approach, type of artificial intelligence, intraoperative application, timing of AI use, training and validation datasets, reported performance metrics, surgeon interaction with AI, influence on intraoperative decision-making, and reported technical and clinical outcomes. Primary outcomes were AI technical performance metrics (e.g., Dice coefficient, IoU, AUC, and F1-score), intraoperative feasibility, and reported clinical outcomes (e.g., recurrent laryngeal nerve/RLN/injury, intraoperative decision impact). Due to heterogeneity of AI performance metrics, no pooled effect measures were calculated. Results are reported using study-specific diagnostic accuracy metrics (e.g., Dice coefficient, AUC, sensitivity, specificity, F1-score). Due to methodological heterogeneity in AI tasks, datasets, and outcome metrics, quantitative synthesis (meta-analysis) was not performed. A structured narrative synthesis was conducted, grouping studies by AI application domain (anatomy recognition, nerve protection, workflow analysis, etc.).

### 2.7. Risk of Bias Assessment

Risk of bias was evaluated using the ROBINS-I tool, and reporting quality was assessed with the STARD 2015 checklist. The results are summarized in Table 1. Overall, studies were at moderate to serious risk of bias, primarily due to retrospective, single-center designs, small sample sizes, and limited external validation. Outcome measurement was often unblinded, and confounding factors and data/frame selection methods were inconsistently addressed. Bias related to intervention classification and deviations was generally low to moderate, highlighting the preliminary nature of current evidence and the need for prospective, standardized validation. Due to the small number of heterogeneous studies, formal assessment of publication bias (e.g., funnel plots) was not performed. Formal certainty-of-evidence assessment (e.g., GRADE) was not conducted due to heterogeneity and absence of comparable outcome measures.

### 2.8. Use of Artificial Intelligence in Figure Creation and Language Editing and Correction

Artificial intelligence-based tools were used to assist in the graphical generation of selected schematic elements within Figure 1 and Figure 2. Specifically, generative design software was employed to create illustrative icons, visual motifs, and background graphical components. The conceptual structure, hierarchy, labeling, scientific content, and overall layout of the figures were designed and defined by the authors. All AI-generated graphical elements were reviewed, modified where necessary, and approved by the authors to ensure scientific accuracy and consistency with the manuscript. No scientific data was generated, altered, or interpreted using AI tools in figure preparation. The use of AI tools did not influence the study design, data extraction, data analysis, or interpretation of results. Artificial intelligence was also used for language editing and correction.

## 3. Results

Several studies evaluated artificial intelligence applications for intraoperative video analysis during esophagectomy, focusing on anatomical recognition, nerve protection, traction detection, and workflow analysis.

Table 2 summarizes key findings in the reviewed literature. Collectively, these studies show that contemporary AI models can achieve performance comparable to expert surgeons in selected tasks and operate at computational speeds compatible with intraoperative video streams (i.e., real-time or near–real-time processing). In some settings, these systems were also able to detect potentially injurious events earlier than conventional intraoperative monitoring. In the context of the included studies, “real-time” refers primarily to computational processing speeds compatible with intraoperative video streams rather than prospective deployment during live surgical procedures.

**Furube et al.** [12] developed a proof-of-concept AI system to detect excessive traction (ET) on the left recurrent laryngeal nerve (RLN) during robot-assisted minimally invasive esophagectomy (RAMIE). The model extended a previously validated anatomical recognition framework and was trained on video frames from 130 RAMIE cases, classifying frames as ET or non-ET and generating a real-time excessive traction risk (ETR) score (0–100%). In an independent evaluation using 10 surgical videos, the system correctly identified 84.4% of ET scenes (38/45). The ETR score correlated with visually assessed traction severity, and in a representative case, ET was detected earlier than changes in nerve integrity monitor (NIM) amplitude, suggesting potential for pre-injury warning.

In a separate study, **Sato et al.** [16] developed a deep learning-based model for real-time detection of the RLN in thoracoscopic esophagectomy videos. The AI system demonstrated superior RLN localization performance compared with general surgeons and performance approaching that of expert esophageal surgeons. The Dice coefficient for AI-based RLN segmentation was 0.58. Quantitative performance metrics indicated clinically relevant detection accuracy with computational inference speeds compatible with real-time video processing.

**Den Boer et al.** [15] evaluated a deep learning model for semantic segmentation of key anatomical structures (azygos vein/vena cava, aorta, and lung) in RAMIE videos. Using 1050 annotated frames (850 for training and 200 for testing), the model achieved median Dice coefficients of 0.79 for the azygos/vena cava, 0.74 for the aorta, and 0.89 for lung segmentation, with real-time inference (~39 frames/s). Performance was comparable to expert annotations for some structures, although greater variability was observed for aortic segmentation, highlighting challenges in reference standard definition.

Another study by **Furube et al.** [13] specifically assessed the diagnostic performance and clinical impact of AI-assisted RLN recognition. A deep learning model trained on 120 RAMIE videos was evaluated on eight external cases. Mean Intersection over Union (IoU) values were 0.40 ± 0.26 for the right RLN and 0.34 ± 0.27 for the left RLN. When AI assistance was provided, surgeons correctly identified the right RLN at the start of lymph node dissection in 81.3% of cases, compared with 46.9% without AI support (*p* = 0.004). During lymph node dissection, IoU values were significantly higher with AI assistance (0.59 ± 0.18 vs. 0.40 ± 0.29; *p* = 0.010), indicating improved anatomical delineation.

**Brandenburg et al. [14]** prospectively investigated the extraction of intraoperative “surgomic” features from robotic esophagectomy videos using machine learning combined with active learning (AL). Across a multicenter dataset of 22 RAMIE videos, 14,004 frames were annotated for ten video-based features related to instruments, bleeding, and anatomical structures. Bayesian ResNet18 models achieved a mean F1-score of 0.75 ± 0.16 across all features, with the highest performance observed for instrument detection. Active learning improved annotation efficiency compared with equidistant sampling, particularly for less frequent instrument classes, while maintaining comparable performance.

Finally, **Takeuchi et al. [17]** reported one of the earliest applications of AI for surgical workflow analysis in RAMIE. A deep learning model was trained to recognize surgical phases from annotated videos, achieving an overall accuracy of approximately 84% using cross-validation. Although the system was applied retrospectively and did not influence intraoperative decision-making, the study demonstrated the technical feasibility of automated workflow recognition in esophageal surgery.

Table 3 summarizes the principal intraoperative applications of artificial intelligence (AI) in esophagectomy, highlighting how contemporary AI systems are being integrated into the operating room to enhance surgical perception, workflow awareness, and decision support. The table categorizes AI use according to application domain, primary function, representative intraoperative tasks, and commonly employed computational methods. Collectively, these applications illustrate the expanding role of computer vision and machine learning in real-time anatomy recognition, surgical phase identification, risk pattern detection, instrument tracking, and augmented intraoperative guidance, with the overarching aim of improving surgical safety, efficiency, and outcomes during complex esophageal procedures.

Overall, included studies primarily evaluated feasibility and technical performance, with limited assessment of downstream clinical outcomes. Although a number of studies reported real-time or near–real-time inference speeds, most models were validated retrospectively using previously recorded surgical videos. In these studies, “real-time” generally referred to computational performance (e.g., frame processing rates compatible with live use) rather than prospective intraoperative deployment with integrated surgeon feedback during active procedures. No included study reported randomized or fully prospective clinical implementation assessing outcome impact during routine surgical workflow. Therefore, current evidence primarily reflects technical real-time capability rather than validated real-time clinical integration. In the context of the included studies, “real-time” means computational processing speeds compatible with intraoperative video streams, typically ranging from approximately 25–40 frames per second or inference times below 50 ms per frame. Such performance thresholds are considered sufficient to avoid perceptible latency during minimally invasive or robotic surgery. However, achieving these frame rates in retrospective testing does not necessarily guarantee seamless integration into live surgical environments, where system latency, hardware constraints, and interface design may influence effective responsiveness.

## 4. Discussion

This systematic review indicates that artificial intelligence-based analysis of intraoperative video during esophagectomy is achievable and may contribute to improved anatomical recognition, risk detection, and procedural understanding [13]. Across multiple studies, AI systems were able to identify critical anatomical structures, detect hazardous mechanical events, and recognize surgical workflow phases in real time or near real time, highlighting the growing potential of AI as an intraoperative support tool in complex esophageal surgery [16].

Several studies focused on protecting the recurrent laryngeal nerve, a structure whose injury is associated with significant postoperative morbidity. Furube et al. extended static anatomical recognition to dynamic risk assessment by developing a real-time AI system capable of detecting excessive traction on the RLN [12]. The reported detection rate of 84.4% and earlier identification of traction compared with nerve integrity monitoring suggest that visually driven AI may enable pre-injury alerts. This represents an important conceptual advance, as the model infers dynamic physiologic risk rather than static anatomy alone. However, the retrospective design, small external test set, and incomplete reporting of reference standards, blinding, and confidence intervals resulted in moderate adherence to the STARD criteria, limiting interpretability and generalizability. Importantly, the effect of such alerts on postoperative RLN palsy rates remains unproven.

Complementary work by Sato et al. demonstrated that deep learning-based RLN detection can outperform general surgeons and approach expert-level recognition during thoracoscopic esophagectomy. These findings support the role of AI in reducing surgeon-dependent variability in anatomical identification. Nonetheless, similar methodological limitations were observed, including incomplete reporting of diagnostic accuracy elements and reliance on retrospective, single-center datasets. While improved RLN recognition is a meaningful surrogate marker, future studies must determine whether these gains translate into measurable reductions in nerve injury and associated complications.

Beyond nerve-specific applications, den Boer et al. showed that AI-driven semantic segmentation can accurately identify major thoracic anatomical structures during robot-assisted minimally invasive esophagectomy, achieving real-time inference and Dice coefficients comparable to expert annotations for selected structures [15]. These findings underscore the feasibility of AI-assisted intraoperative guidance. However, performance variability between structures and inter-annotator differences highlights persistent challenges in defining reliable reference standards, a recurring limitation across surgical AI studies.

More broadly, the reviewed literature revealed substantial heterogeneity in evaluation metrics, validation strategies, and reporting quality. Many studies relied on overlap-based metrics such as the Dice coefficient or Intersection over Union, which do not directly capture clinical safety or patient-centered outcomes. Bridging the gap between technical accuracy and clinical utility requires carefully designed prospective studies. These studies should include predefined patient-centered endpoints. Future trials should evaluate whether AI-assisted anatomy recognition or risk detection translates into measurable reductions in clinically relevant complications, such as recurrent laryngeal nerve palsy, anastomotic leakage, intraoperative blood loss, or operative time. Randomized or controlled prospective study designs comparing AI-assisted versus conventional surgery would provide higher-level evidence regarding efficacy. In addition to complication rates, workflow metrics, surgeon cognitive load, and decision-modification frequency should be assessed to determine how AI integration influences intraoperative behavior. Only through such outcome-driven validation can technical performance metrics be meaningfully linked to improvements in surgical safety and patient outcomes. Validation cohorts among the available studies were frequently small. External or prospective validation was uncommon.

An additional concern relates to domain shift. Model performance may deteriorate when applied to data that differ from the original training environment. Most included studies relied on single-center datasets using specific imaging systems and surgical workflows. Variations in lighting, video resolution, tissue characteristics, and surgeon technique may affect robustness. Without multicenter training and out-of-sample validation, generalizability remains uncertain.

Future research should prioritize collaborative data sharing and multicenter validation to ensure that AI models maintain stable performance across diverse surgical environments. Incomplete reporting of blinding, missing data handling, and precision estimates further constrained reproducibility, as reflected by moderate STARD scores across studies. The presence of serious risk of bias in several studies, particularly in the domain of outcome measurement, warrants careful interpretation of reported performance metrics. In many cases, reference annotations were performed retrospectively and without blinding, potentially introducing observer bias and overestimation of model accuracy. Furthermore, the absence of standardized ground-truth definitions for anatomical segmentation may have influenced overlap-based metrics such as Dice coefficient and IoU. These methodological limitations suggest that reported performance values should be interpreted as preliminary technical estimates rather than definitive indicators of clinical effectiveness.

Emerging work in surgomics and workflow analysis expands the scope of intraoperative AI beyond anatomy recognition. Brandenburg et al. demonstrated that active learning can reduce annotation burden while maintaining robust performance in multicenter surgical video datasets, representing a critical step toward scalable AI development [14]. Similarly, Takeuchi et al. established the feasibility of automated surgical phase recognition, providing foundational context-awareness upon which future real-time guidance and decision-support systems may be built [17]. However, both approaches remain primarily technical, with limited linkage to clinical outcomes.

Collectively, the available evidence suggests that AI-based systems have the capacity to augment intraoperative perception during esophagectomy by improving anatomical recognition, detecting procedural risk, and contextualizing surgical workflow [18,19,20]. Nevertheless, substantial methodological and translational barriers remain. Future research should prioritize prospective, multicenter validation; standardized and transparent reporting frameworks (including STARD, TRIPOD-AI, and emerging surgical AI guidelines); and evaluation of clinically meaningful endpoints such as nerve injury, complication rates, and operative safety [21]. Until these gaps are addressed, AI systems in esophagectomy should be viewed as promising adjuncts rather than fully validated clinical tools.

Given the sensitivity of surgical video data and regulatory constraints surrounding patient privacy, federated learning represents a promising strategy for future multicenter collaboration. Federated learning enables model training across institutions without transferring raw patient data, allowing decentralized data utilization while preserving confidentiality. Such approaches may help mitigate domain shift, improve generalizability, and facilitate large-scale validation of intraoperative AI systems in esophagectomy without compromising data security.

Bearing in mind the early-phase and heterogeneous nature of all included studies, overall certainty of evidence is considered low to moderate.

## 5. Limitations

This review has several limitations. First, the available evidence is limited by the early-stage nature of research in this field, with most included studies being retrospective, single-center investigations with small validation cohorts. Second, substantial heterogeneity in artificial intelligence methodologies, datasets, outcome definitions, and reporting standards prevented quantitative synthesis and limited cross-study comparability. Third, the reliance on institution-specific datasets may restrict generalizability across surgical environments. Finally, although comprehensive search strategies were used, the possibility of publication bias cannot be excluded. These limitations highlight the need for larger prospective multicenter studies with standardized reporting and outcome evaluation.

## 6. Conclusions

Artificial intelligence-based intraoperative video analysis in esophagectomy represents an emerging approach that may enhance anatomical recognition, risk detection, and procedural understanding. Current evidence indicates that AI can support identification of critical structures such as the recurrent laryngeal nerve, detect hazardous intraoperative events, and contextualize surgical workflow in real time. However, available studies are largely proof-of-concept, retrospective, and single-center, with limited independent validation and scarce assessment of clinical outcomes. Before routine clinical implementation, prospective, multicenter studies evaluating patient-centered endpoints and standardized reporting frameworks are required. In the interim, AI should be considered a complementary intraoperative tool with potential to augment, rather than replace, surgeon expertise.

## Figures and Tables

**Table 1:** Assessing Risk of Bias and reporting quality for the final study selection.

Title	Furube et al. (2025) [12]	Furube et al. (2024) [13]	Brandenburg et al. (2023) [14]	den Boer et al. (2023) [15]	Sato et al. (2022) [16]	Takeuchi et al. (2022) [17]
**Bias due to confounding**	serious	moderate	moderate	moderate	moderate	moderate
**Bias in selection of participants**	moderate	moderate	moderate	moderate	moderate	moderate
**Bias in classification of intervention**	moderate	low	low	low	low	low
**Bias due to deviations from intended intervention**	low	low	low	low	low	low
**Bias due to missing data**	moderate	moderate	moderate	moderate	moderate	low
**Bias in measurement of outcomes**	serious	serious	serious	serious	moderate	moderate
**Overall risk of bias**	serious	serious	moderate	moderate	moderate	moderate
**STARD 2015 Checklist Assessment**	63% (17/27)	59% (16/27)	67% (18/27)	60% (18/30)	70% (19/27)	68% (17/25)

**Table 2:** Summary of published studies evaluating intraoperative artificial intelligence applications in esophagectomy, including AI task focus and key performance metrics.

Study (Year)	AI Task	Key Performance Metrics	Model Architecture/Framework
**Furube et al. (2025) [12]**	Real-time detection of excessive traction on RLN	Correct detection of unintended nerve traction: **84.4%**; Excessive traction risk (ETR) score correlated with traction degree; AI detected ET earlier than NIM signal in representative case (pre-injury potential)	Deep learning-based convolutional neural network (CNN) built upon previously developed anatomical recognition framework; frame-level binary classification model for excessive traction detection with real-time risk score generation
**Furube et al. (2024) [13]**	RLN segmentation and recognition metrics	AUC: **0.92** (left), **0.88** (right); Dice: **0.72**; Sensitivity: **0.86**; Specificity: **0.89**; IoU: **0.40 ± 0.26** (right), **0.34 ± 0.27** (left); surgeon assistance improved RLN recognition rates and IoU with AI assistance	CNN-based semantic segmentation model for RLN recognition (deep learning segmentation framework; supervised training on annotated RAMIE frames)
**Brandenburg et al. (2023) [14]**	Surgomic feature recognition (Active Learning)	Mean F1-score: **0.75 ± 0.16** (all features); instrument detection F1: **0.80 ± 0.17**; inter-rater agreement κ > **0.82**; AL improved rare instrument sample selection and performance vs. EQS	Bayesian ResNet18 backbone combined with Active Learning (AL) framework for surgomic feature classification
**den Boer et al. (2023) [15]**	Anatomical structure segmentation (Bayesian NN)	Median Dice: **0.79** (azygos/vena cava), **0.74** (aorta), **0.89** (lung); algorithm comparable to expert annotations; inference time ~**0.026 s/frame (39 Hz)**	Bayesian convolutional neural network for semantic segmentation (uncertainty-aware deep learning architecture)
**Sato et al. (2022) [16]**	Recurrent laryngeal nerve (RLN) segmentation	Dice coefficient: 0.58 (AI) vs. 0.62 (expert) vs. 0.47 (general surgeons); AI performance superior to general surgeons (*p* = 0.019)	U-Net-based deep learning semantic segmentation model for RLN detection
**Takeuchi et al. (2022) [17]**	Surgical phase recognition	Overall accuracy: **84%**; precision: ~**0.84**; per-phase recall: **58–93%**	CNN-based model combined with temporal sequence modeling (CNN + LSTM) for surgical phase recognition

**Table 3:** Intraoperative applications of artificial intelligence in esophagectomy, categorized by application domain, primary function, representative intraoperative use, and typical AI methods.

AI Application Domain	Primary Function	Representative Intraoperative Use	Typical AI Methods
**Anatomy detection and recognition**	Identification and delineation of critical structures	Real-time recognition of RLN, aorta, azygos vein, lung during dissection	CNN-based deep learning, semantic segmentation, transfer learning
**Surgical phase recognition**	Temporal classification of procedural steps	Automated identification of operative phases to provide contextual awareness	Deep learning with CNNs and temporal models (e.g., RNN/LSTM)
**Pattern and event detection**	Detection of predefined intraoperative risk patterns	Identification of excessive traction, bleeding, smoke, or unsafe tissue handling	Deep learning classification models, CNNs, active learning
**Instrument detection and tracking**	Recognition and localization of surgical tools	Real-time tracking of instruments to infer surgical intent and motion	Computer vision, CNN-based object detection
**Intraoperative guidance and decision support**	Augmented cognition and risk mitigation	Visual overlays, alerts for nerve traction, anatomy highlighting	AI-augmented computer vision, augmented reality, ML-based risk models

## Data Availability

The data supporting the findings of this systematic review, including the search strategy and screening documentation, are available in the PROSPERO registry under registration number CRD420261295799.

## Supplementary Materials

The following supporting information can be downloaded at: https://www.mdpi.com/article/10.3390/jcm15062169/s1, Table S1: PRISMA Checklist; Table S2: Assessing Risk of Bias and reporting quality for the final study selection; Search strategy; List of articles from each database; Spreadsheet after article screening.

## Abbreviations

The following abbreviations are used in this manuscript:

AI	Artificial intelligence
MIE	Minimally invasive esophagectomy
RAMIE	Robot-assisted minimally invasive esophagectomy
ET	Excessive traction
RLN	Recurrent laryngeal nerve
NIM	Nerve integrity monitor
CNN	Convolutional neural network
RNN	Recurrent neural network
LSTM	Long short-term memory
ML	Machine learning
AUC	Area under the curve
IoU	Intersection over Union
