# Supplementary material for: Artificial Intelligence in Esophagectomy: A Systematic Review
Source: J Clin Med. 2026 Mar 12;15(6):2169. doi: 10.3390/jcm15062169 (PMC13027133; doi:10.3390/jcm15062169)
Supplement: Supplementary file 1 [file jcm-15-02169-s001.zip › Articles data and results.pdf]

Study (Year)	AI Task	Key Performance Metrics
**Furube et al. (2025)**	Real-time detection of excessive traction on RLN	Correct detection of unintended nerve traction: **84.4%**; ETR value correlated with traction degree; AI detected ET earlier than NIM signal in representative case (pre-injury potential)
**Furube et al. (2024)**	RLN segmentation and recognition metrics	AUC: **0.92** (left), **0.88** (right); Dice: **0.72**; Sensitivity: **0.86**; Specificity: **0.89**; IoU: **0.40 ± 0.26** (right), **0.34 ± 0.27** (left); surgeon assistance improved RLN recognition rates and IoU with AI assistance
**Brandenburg et al. (2023)**	Surgomic feature recognition (Active Learning)	Mean F1-score: **0.75 ± 0.16** (all features); instrument detection F1: **0.80 ± 0.17**; inter-rater agreement κ > **0.82**; AL improved rare instrument sample selection and performance vs EQS
**den Boer et al. (2023)**	Anatomical structure segmentation (Bayesian NN)	Median Dice: **0.79** (azygos/vena cava), **0.74** (aorta), **0.89** (lung); algorithm comparable to expert annotations; inference time ~ **0.026 s/frame (39 Hz)**
**Sato et al. (2022)**	Recurrent laryngeal nerve (RLN) segmentation	Dice coefficient: 0.58 (AI) vs 0.62 (expert) vs 0.47 (general surgeons); AI performance superior to general surgeons (p = 0.019)
**Takeuchi et al. (2022)**	Surgical phase recognition	Overall accuracy: **84%**; precision: **~0.84**; per-phase recall: **58–93%**
