# Supplementary material for: Artificial Intelligence in Esophagectomy: A Systematic Review
Source: J Clin Med. 2026 Mar 12;15(6):2169. doi: 10.3390/jcm15062169 (PMC13027133; doi:10.3390/jcm15062169)
Supplement: Supplementary file 1 [file jcm-15-02169-s001.zip › Intraoperative Applications of Artificial Intelligence in Esophagectomy.pdf]

AI Application Domain	Primary Function	Representative Intraoperative Use	Typical AI Methods
**Anatomy detection and recognition**	Identification and delineation of critical structures	Real-time recognition of the RLN, aorta, azygos vein, and lung during dissection	CNN-based deep learning, semantic segmentation, and transfer learning
**Surgical phase recognition**	Temporal classification of procedural steps	Automated identification of operative phases to provide contextual awareness	Deep learning with CNNs and temporal models (e.g., RNN/LSTM)
**Pattern and event detection**	Detection of predefined intraoperative risk patterns	Identification of excessive traction, bleeding, smoke, or unsafe tissue handling	Deep learning classification models, CNNs, and active learning
**Instrument detection and tracking**	Recognition and localization of surgical tools	Real-time tracking of instruments to infer surgical intent and motion	Computer vision and CNN-based object detection
**Intraoperative guidance and decision support**	Augmented cognition and risk mitigation	Visual overlays, alerts for nerve traction, and anatomy highlighting	AI-augmented computer vision, augmented reality, and ML-based risk models
