# Supplementary material for: Artificial Intelligence in Esophagectomy: A Systematic Review
Source: J Clin Med. 2026 Mar 12;15(6):2169. doi: 10.3390/jcm15062169 (PMC13027133; doi:10.3390/jcm15062169)
Supplement: Supplementary file 1 [file jcm-15-02169-s001.zip › Search strategy.pdf]

Pubmed search strategy:

(( esophagectomy[Title/Abstract] OR oesophagectomy[Title/Abstract] OR "Ivor-Lewis" OR "Mckeown" OR oesophagectomy[Title/Abstract] ) AND ( "artificial intelligence"[Title/Abstract] OR "machine learning"[Title/Abstract] OR "deep learning"[Title/Abstract] OR "computer vision"[Title/Abstract] OR "neural network*"[Title/Abstract] OR "anatomy detection"[Title/Abstract] ) AND ( "intraoperative"[Title/Abstract] OR "intra-operative"[Title/Abstract] OR "real-time"[Title/Abstract] OR "surgical guidance"[Title/Abstract] ) ) AND (( esophagectomy[Title/Abstract] OR "Ivor-Lewis" OR Mckeown OR oesophagectomy[Title/Abstract] ) AND ( "artificial intelligence"[Title/Abstract] OR "machine learning"[Title/Abstract] OR "deep learning"[Title/Abstract] OR "computer vision"[Title/Abstract] OR "neural network*"[Title/Abstract] OR "anatomy detection"[Title/Abstract] ) AND ( "intraoperative"[Title/Abstract] OR "intra-operative"[Title/Abstract] OR "real-time"[Title/Abstract] OR "surgical guidance"[Title/Abstract] ))

Scopus search strategy:

(TITLE-ABS-KEY("esophagectomy" OR "oesophagectomy") AND TITLE-ABS-KEY("artificial intelligence" OR "machine learning" OR "deep learning" OR "computer vision" OR "neural network*"))

Web of Science search strategy:

TS=(esophagectomy OR oesophagectomy)

AND

TS=("artificial intelligence" OR "machine learning" OR "deep learning" OR "computer vision" OR "neural network*")
